# Barrettides: A Peptide Family Specifically Produced by the Deep-Sea Sponge *Geodia barretti*

**DOI:** 10.1021/acs.jnatprod.1c00938

**Published:** 2021-12-07

**Authors:** Karin Steffen, Quentin Laborde, Sunithi Gunasekera, Colton D. Payne, K. Johan Rosengren, Ana Riesgo, Ulf Göransson, Paco Cárdenas

**Affiliations:** † Pharmacognosy, Department of Pharmaceutical Biosciences, Biomedical Centre, Uppsala University, Husargatan 3, 751 23 Uppsala, Sweden; ‡ School of Biomedical Sciences, The University of Queensland, Brisbane, QLD 4072, Australia; ¶ Department of Life Sciences, The Natural History Museum, Cromwell Road, London SW7 5BD, United Kingdom; § Department of Biodiversity and Evolutionary Biology, Museo Nacional de Ciencias Naturales−CSIC, Calle José Gutiérrez Abascal 2, 28006, Madrid, Spain

## Abstract

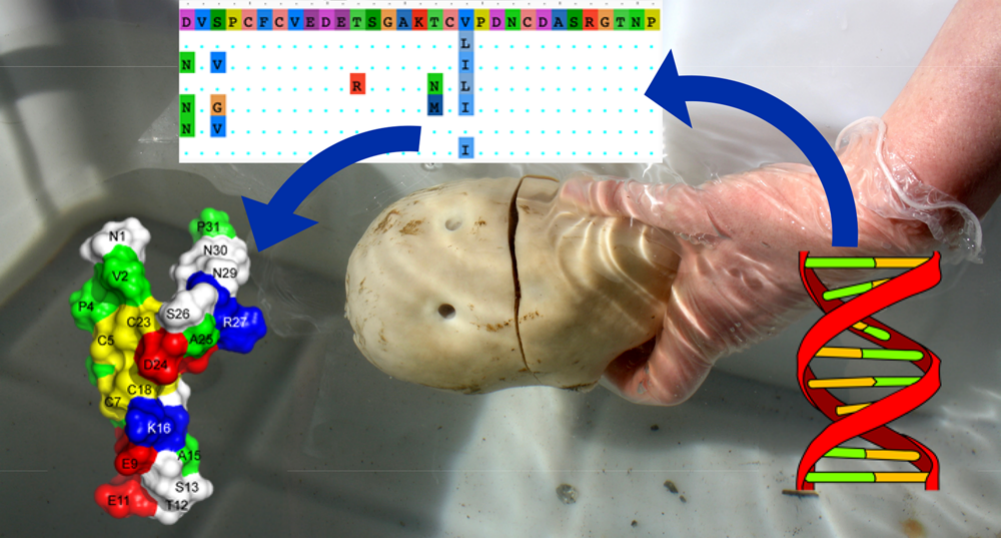

Natural product discovery by isolation and structure elucidation is a laborious task often requiring ample quantities of biological starting material and frequently resulting in the rediscovery of previously known compounds. However, peptides are a compound class amenable to an alternative genomic, transcriptomic, and *in silico* discovery route by similarity searches of known peptide sequences against sequencing data. Based on the sequences of barrettides A and B, we identified five new barrettide sequences (barrettides C–G) predicted from the North Atlantic deep-sea demosponge *Geodia barretti* (Geodiidae). We synthesized, folded, and investigated one of the newly described barrettides, barrettide C (NVVPCFCVEDETSGAKTCIPDNCDASRGTNP, disulfide connectivity I–IV, II–III). Co-elution experiments of synthetic and sponge-derived barrettide C confirmed its native conformation. NMR spectroscopy and the anti-biofouling activity on larval settlement of the bay barnacle *Amphibalanus improvisus* (IC_50_ 0.64 μM) show that barrettide C is highly similar to barrettides A and B in both structure and function. Several lines of evidence suggest that barrettides are produced by the sponge itself and not one of its microbial symbionts.

Marine sponges are recognized as a prolific source of new marine natural products (NPs).^1−3^ However, these sponge NPs are frequently found to be produced by their associated microbes.^4−12^*Geodia barretti* Bowerbank,^13^ 1858 is a species of North Atlantic deep-sea demosponge well studied since the 1980s for its natural products.^14^ To date, 12 small molecules have been reported to be associated with the sponge, namely, herbipolin,^15−17^ barettin,^18^ 8,9-dihydrobarettin,^19^ the diketopiperazine cyclo(Pro-Arg) and 8,9-dihydro-8-hydroxybarrettin,^20^ bromobenzisoxalone barettin,^21^ 6-bromoconicamin and 6-bromo-8-hydroxyconicamin,^22^ geobarrettins A, B, and C, and L-6-bromohypaphorine,^23^ with a range of bioactivities. In addition, two bioactive peptides, barrettides A and B, have been isolated from *G. barretti*.^24^

The barrettide peptides are each composed of 31 amino acid residues, two disulfide bonds (Cys5–Cys23 and Cys7–Cys18), and two antiparallel β-strands, which creates an overall β-hairpin structure. Barrettides A and B were found to inhibit biofouling by the bay barnacle *Amphibalanus improvisus* (Darwin, 1854).^25^ Carstens et al.^24^ further noted the presence of two additional peptidic masses at low abundance, [M + 2H]^2+^ 1619 and 1648. Isolation and structure elucidation of these low-abundance peptides would require a substantial amount of biomass, making this a nonviable strategy.

An alternative for discovering new peptides is straightforward BLAST searches using known peptide sequences as queries. These searches can be performed on transcriptomes that are abundant in sequencing databases. Such a data survey has the potential to address key challenges in natural product chemistry: (1) Sequencing data can be instrumental in identifying the true producer of (sponge) NPs.^5,7−11^ (2) By discovering new similar sequences, diversity of previously described peptides may be expanded and can be placed in the context of a larger family of compounds. (3) Appropriate sequencing data can be used to study the specificity or taxonomic distribution of an NP by establishing whether the compound is restricted to a single species or present in related taxa.

Here, our rationale was to investigate these three key challenges with respect to the barrettides, (i.e., their diversity, distribution, and origin) using high-throughput sequencing data of sponges in general and *G. barretti* in particular. As a proof of concept and to assess the validity of a predicted sequence, we synthesized and folded the predicted sequence named barrettide C, demonstrated its presence in the sponge, elucidated its structure using NMR spectroscopy, and tested its antifouling activity.

## Results and Discussion

### *In Silico* Discovery

We gathered, processed, and refined a collection of sequencing data sets representative of the sponge phylum as a whole and for the species *G. barretti* in particular (Supporting Information Table S1). For *G. barretti*, transcriptomes were assembled from raw data for seven individuals. Quality filtering after assembly decreased the number of retained gene sets (and thus transcripts) quite drastically in some cases (Gb01: from 136 297 to 102 692, Gb02: from 232 941 to 196 679, Gb03: from 113 124 to 96 728, Gb04: from 156 088 to 110 425, Gb05: from 160 210 to 94 448, Gb06: from 175 753 to 113 976, Gb07: from 89 358 to 8). While the number of genes is still excessive and should be taken with caution, it is to be expected for *de novo* transcriptome assemblies. For comparison, the number of genes predicted from sponge whole genomes range from 32 309 in *Sycon ciliatum*(26) to 40 112 in *Amphimedon queenslandica*.^27,28^ For the BLAST searches in *G. barretti*, in addition to the transcriptomes mentioned above, an assembled metatranscriptome^29^ and an in-house draft genome (unpublished) were included. The draft genome of *G. barretti* has a length of 144.8 Mb across 4576 contigs and is estimated to be 93.9% complete with only 4.2% duplicated single orthologous genes (BUSCO v.3.0.2b). Beyond *G. barretti*, readily assembled transcriptomes from 63 additional sponge species were collected. Combined, these data sets include representatives of all four classes of sponges and add up to five Hexactinellida, four Calcarea, three Homoscleromorpha, and 52 Demospongiae (covering 15 out of the 22 current valid orders).

**Figure 1:**
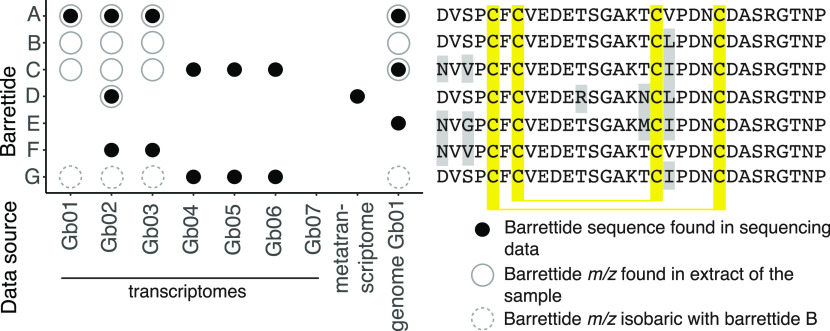
Identification of barrettides across different *G. barretti* samples. Full black circles: barrettide sequence found in respective sequencing data, open gray circles: barrettide *m*/*z* found in metabolic profile of the same sample. Dashed gray circles: *m*/*z* isobaric with barrettide B. The corresponding amino acid sequences are shown to the right, and amino acid differences to barrettide A are highlighted with gray shading. Gb07 did not yield any hits due to the quality filtering process removing most of the assembled transcripts.

In total, searching *G. barretti* sequencing data yielded five new sequences, tentatively named barrettides C–G (Supporting Information Tables S2, S3). The amino acid sequences of the predicted barrettides and their distribution across the sequencing data are shown in Figure 1; their *m*/*z* ions in Table 1. These sequences have one to four amino acid changes compared to the prototypic barrettide A. After assembly of the raw data from the metatranscriptome,^29^ we found one full-length barrettide sequence (D), contrary to previous findings which report a lack of barrettide sequences in the unassembled data.^24^ The previously published barrettide B was not recovered from the sequencing data. For barrettides A, C, D, F, and G, transcripts contained the barrettide sequence in an open reading frame (ORF). The draft genome is derived from the same sample as the transcriptome Gb01 and contains two barrettides (C, E) not found in the transcriptomes. This could be due to these barrettides not being expressed at the time of collection or due to an assembly error. The fact that we do not recover barrettide B in DNA sequencing data of the *G. barretti* samples but another isobaric sequence (barrettide G) is not due to a mistake in the original publication.^24^Supporting Information Tables S5 and S6 present the original amino acid analysis data as well as complementary information from peptide sequencing confirming the sequence of barrettide B is valid and correct. However, the validation of barrettide G predicted from the transcriptome would require isolation and amino acid analysis, NMR, or other methods able to distinguish between isobaric amino acids.

**Table 1:** Barrettide *m/z* with Cysteines Oxidized, Inferred from Their Respective Amino Acid Sequences and Mass Error from Experimental Data

barrettide	[M + H]^+^	[M + 2H]^2+^	[M + 3H]^3+^	[M + 4H]^4+^	experimental	mass error
A	3213.2864	1607.1468	1071.7670	804.0771	3212.307	2.4904
B	3227.3020	1614.1547	1076.4389	807.5810	3226.3236	2.6656
C	3238.3544	1619.6808	1080.1230	810.3441	3237.3762	2.8418
D	3295.3507	1648.1790	1099.1218	824.5931	3294.3784	4.3711
E	3226.3003	1613.6538	1076.1049	807.3305		
F	3224.3388	1612.6730	1075.4511	806.8401		
G	3227.3020	1614.1547	1076.4389	807.5810		

In all other species’ transcriptomes (*n* = 63), the BLAST searches and subsequent filtering revealed 15 sequences somewhat similar to barrettides A and B from 11 sponge transcriptomes of Demosponges and Hexactinellida (Supporting Information Table S4). Given the conserved positions of cysteine residues, these hits could represent potential hairpin peptides in these sponge species. Although these searches included transcriptomes of four close relatives of *G. barretti* (*G. atlantica*, *G. macandrewi*, *G. phlegraei*, *G. hentscheli*), full-length peptide sequence hits with high sequence similarity were only recovered from *G. barretti* sequencing data.

Barrettide sequences are directly coded in the DNA/RNA akin to the peptide NP class of ribosomally synthesized and post-translationally modified peptides (RiPPs), one of two classes of peptide NPs.^30^ However, barrettides do not contain any of the modifications characteristic for RiPPs. Yet, with the obtained sequences at hand, we searched for features analogous to the modular precursor peptides of RiPPs, which can contain a signal domain, leader and core peptide, and recognition sequences.^30^ The barrettide sequences and peptides presented here (Figure 1, Table 1) would correspond to the mature RiPPs derived from the core peptide. The only post-translational modifications present in barrettides are the formation of two disulfide bonds (Cys5–Cys23 and Cys7–Cys18). As for RiPPs, the barrettide ORFs are consistently predicted to start with a signal peptide of 19 residues, MA**T**KVALL**V**VSALIAVAAA/MA**I**KVALL**A**VSALIAVAAA. A region of variable length (either short (19) or long (105 or 112 residues)) is present between the predicted signal and the core peptide and could therefore constitute a segment analogous to the leader peptide (Supporting Information Table S3). At the end of the putative leader peptide, i.e., just before the start of the core barrettide, there is an Ala residue present in all sequences. In the framework of RiPPs, specific enzyme cleavage sites should be present in the sequence to cleave the different modules from the precursor peptide. In eukaryotes, signal peptidase I (SPase I, EC 3.4.21.89) typically cleaves off the signal peptide,^31^ while other sequence-specific peptidases cleave the leader peptide from the core peptide. For the barrettides, proteinase K (EC 3.4.21.62 and 3.4.21.64) and Asp-N endopeptidase (+ N-terminal Glu) (EC 3.4.24.33) were predicted to cleave between the Ala residue and the core peptide. Of these enzymes, only the EC numbers for SPase I yielded matches in the transcriptome annotation of Gb01. However, there are many other peptidases (EC 3.4.) annotated. Finally, after the last/C-terminal residue of the core peptide, a stop codon (TGA) is present in all sequences. In theory, the translation machinery would dissociate from the growing peptide when reaching the stop codon, and thus we have no C-terminal recognition sequences. Yet, at the RNA level, a conserved 3′ UTR (untranslated region, approximately 33 nucleotides) is present. At this point, we cannot speculate about its function as BLAST searches of 3′ (and 5′ UTRs) against the NCBI GenBank nucleotide collection did not result in any significant similarity.^32^

### What Evidence Do We Have for the Origin of the Barrettides?

Given the compositional complexity of sponges with their associated microbes (considered contamination in the sponge transcriptomes), evaluating evidence for either a prokaryotic (microbe) or eukaryotic (sponge) origin of the barrettides requires cautious assessment of the multiple levels of evidence. In less complex (i.e., uncontaminated) tissue samples, the presence of a transcript could be deemed sufficient evidence for the producer of a transcript and thus a compound. However, we detected contamination in sponge transcriptomes by a bimodal distribution of GC content as indicated by fastQC despite RNA sequencing library preparation steps to decrease the abundance of prokaryotic transcripts. Thus, the mere presence of a transcript is not sufficient evidence to implicate the sponge as its producer. We further investigated the transcripts for prokaryotic or eukaryotic characteristics. A sign of a mature eukaryotic transcript is a poly-A tail, i.e., “A”s (adenines) at the end of the nucleotide sequence. In two barettide transcripts from Gb04, we observed short poly-A tails. Investigating the specific transcript quality, by mapping back raw sequencing reads, did not strongly support the poly-A tails, as there was little continuous coverage of the transcript toward the tail of the sequences. In addition, poly-A tails are also found elsewhere, i.e., in bacteria and archaea (for degradation purposes).^33^ Therefore, this line of evidence seems inconclusive as to whether or not the sponge is the producer of the transcripts. Introns, noncoding regions in transcripts that are removed prior to translation, are typically regarded as a eukaryotic feature, although there are two types of introns (“group I” and “group II”) found in prokaryotes.^34−36^ Aligning several isoforms (e.g., five isoforms hits for barrettide A in Gb03 or six isoforms for barrettide G in Gb04) did reveal several introns. These introns (<100 up to 400 nucleotides in length) did not yield any significant BLAST hits against the GenBank nucleotide collection. In our opinion, a true prokaryotic group I intron would have produced significant hits due to the conserved domains. Further, the introns found in our transcripts are too short to be prokaryotic group II introns (500–800 bp). Therefore, on the basis of intron length and lack of sequence similarity, we hypothesized the introns to be indicators of eukaryotic transcripts. Despite being at the draft stage, the genome, which has undergone *in silico* contamination removal, also contains genes for barrettides. We also checked metabolic profiles of *G. barretti* samples, acquired in the framework of an ecological study of sponge-derived chemistry (Steffen et al., unpublished), for barrettide C (Supporting Information Figure S1) and found a similar pattern to that for barrettides A and B, i.e., individual variation in expression not dependent on sample depth. The peptide thus seems constitutively produced, which could point toward the sponge as its producer. With this combined evidence at hand (eukaryotic introns, barrettide sequences present in the genome, constitutive expression), we think it is reasonable to suggest that sponges are the producers of the barrettides. This would make barrettides the first sponge-derived peptide NP attributed to the sponge and not a prokaryotic microbial symbiont.

Generally, sponges are known as a prolific source of natural products.^1−3,37^ For natural products typically considered primary metabolites, such as sterols,^38^ sponges have been established as the producers.^2,39−41^ However, many sponge-derived natural products typically considered as “secondary” or “specialized” metabolites have been found to be of microbial origin.^4−12^ For various “secondary” NPs, localization studies show association with sponge cells.^42,43^ Further, in some cases, sphaerulous cells loaded with natural products were found to be exuded by sponges (e.g., in *Crambe crambe* and *Aplysina fistularis*).^44,45^ However, *Haliclona* sp. was found to harbor intracellular symbionts producing specialized metabolites, which would refute the underlying assumption that sponge cell localization is sufficient evidence to implicate the sponge as the producer of the metabolite.^7,46^ This finding highlights the importance of determining the genetic proof of NP production. Also, compounds could be taken up by sponge cells after being produced by a microbe. Alternative to DNA sequencing, fluorescence *in situ* hybridization methods have been used to establish the bacterial origin of a sponge-derived peptide.^47^

### Detecting Barrettide Presence by LC-MS

Based on the amino acid sequences and disulfide connectivity, the calculated [M + 2H]^2+^ of barrettides C and D (1619.6808 and 1648.1790, respectively) matched the masses observed in a previous extract.^24^ To follow up on barrettide reports from the transcriptomes, we searched metabolic profiles of samples Gb01, Gb02, and Gb03, observing *m*/*z* from barrettide A to C, A to D, and A to C, respectively (Figure 1). We do not have extracts for samples Gb04, Gb05, Gb06, and Gb07 and cannot corroborate the presence of barrettides by their *m*/*z*. The detection of predicted barrettide G would require additional methods for verification, as it is isobaric with barrettide B; the sequences only differ by an interchange of leucine/isoleucine. Beyond *G. barretti*, we did not observe barrettides *m*/*z* in extracts of *G. atlantica* (UPSZMC 78293), *G. macandrewii* (UPSZMC 78255), *G. phlegraei* (UPSZMC 167249), or *G. hentscheli* (UPSZMC 78266) corroborating the limitation of barrettides to *G. barretti* observed in the transcriptomic data.

The comparison of transcriptome surveys and MS data showed that the two kinds of data capture different aspects of the biology and physiological output of the organisms. Sequences retrieved from transcriptomes were confirmed by their corresponding *m*/*z* (barrettide A in Gb01, Gb02, and Gb03 and barrettide D in Gb02). However, some barrettides predicted from the transcriptome were not found in the MS data (barrettide F in Gb02 and Gb03) and *vice versa* (barrettides B and C in Gb01, Gb02, and Gb03). A number of plausible mechanisms could explain these discrepancies starting from technical reasons, e.g., sequencing errors or misassemblies in the transcriptome, sensitivity of MS analyses to biological explanations such as modifications of transcripts (degradation), post-translational modifications of the barrettide precursor peptide, or storage/accumulation of previously produced barrettides in the sponges, or simply lack of expression. Barrettide E was only present in the genome and appeared not to be expressed at either the transcriptome or the metabolome level.

### Peptide Synthesis and LC-MS

**Figure 2:**
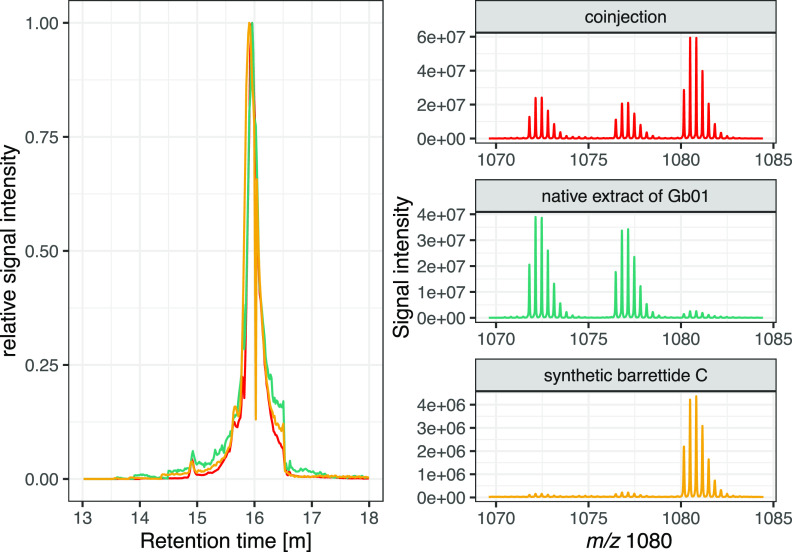
Left: Selected ion mass chromatograms for the *m*/*z* 1080 [M + 3H]^3+^ of barrettide C. The synthetic peptide (yellow) has the same retention time (*t*_R_) as the *m*/*z* observed in the native chemical profile (turquoise). Likewise, the native chemical extract spiked with synthetic barrettide C (red) only shows one peak at the same *t*_R_. Right: Isotopic patterns show a signal increase in the spiked native extract both in absolute terms and relative to the intensities of barrettide A ([M + 3H]^3+^ 1071) and B ([M + 3H]^3+^ 1076).

Barrettide C was chosen for experimental validation of the sequence predictions, as it was present in all investigated metabolic profiles. The peptide was synthesized on a solid support using standard Fmoc [*N*-(9-fluorenyl)methoxycarbonyl] chemistry. As the C-terminal Pro is prone to diketopiperazine formation that leads to cleavage of the emerging peptide chain from the resin, we chose 2-chlorotrityl chloride (2-CTC) as the resin to avoid this reaction. Pro was coupled as the first residue, and the peptide was assembled by manual synthesis. As in previous work, we incorporated a dipeptide, in our case Fmoc-Ser(tBu)-(Dmb)Gly-OH, at positions 18 and 19 to prevent peptide aggregation and improve synthesis yield.^24^ Using this approach, the peptide could be synthesized in good yields and was subsequently successfully folded and purified. The peptide had good solubility in water in both reduced and oxidized form. A coelution experiment was performed by LC-MS comparing synthetic barrettide C with a sponge extract containing native barrettide C (Gb01) and the native extract spiked with the synthetic barrettide C (Figure 2). Retention times and isotopic patterns confirmed that the synthetic barrettide C and the native compound were the same.

### Structure Determination of Barrettide C and Comparison to Barrettide A

**Figure 3:**
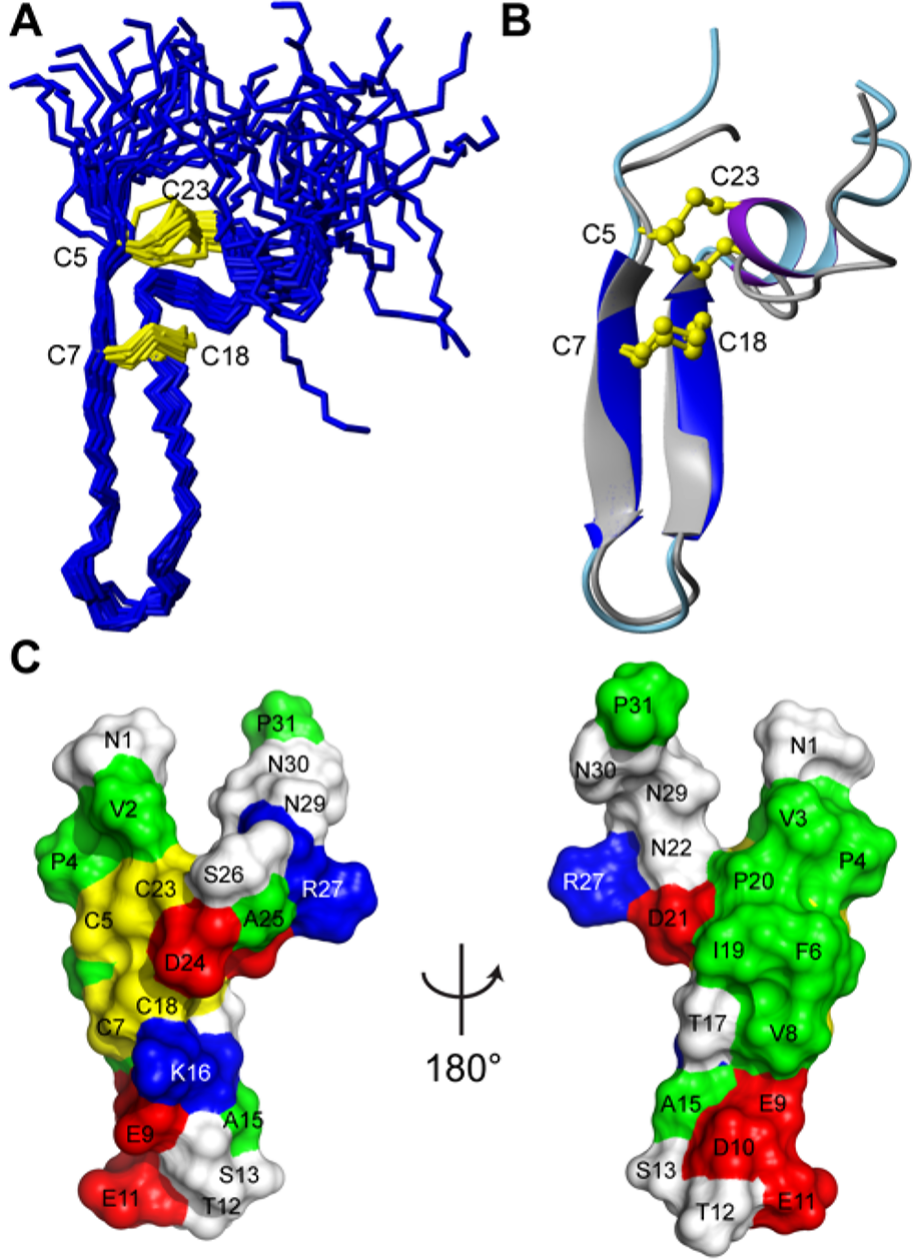
Solution NMR structure of barrettide C and comparison to barrettide A. (A) Superimposed structural ensemble of barrettide C in stick format, with disulfide bonds in yellow. (B) Superimposition of barrettide C (dark blue β-sheet, light blue backbone, and purple α-helix) and barrettide A (gray β-sheet and backbone) in ribbon format with ball-and-stick disulfide bonds in yellow. This superimposition highlights the conserved β-hairpin structure of the barrettides. (C) Surface representation of barrettide C. Colors are used to denote residue side chain properties: negatively charged (red), positively charged (blue), hydrophobic (green), disulfide (yellow), and polar (white). Individual amino acids are also labeled for orientation.

Previously discovered sequences of barrettides are highly similar with most observed amino acid changes being conservative (Figure 1). Thus, one would expect their 3D structures to be highly conserved. To confirm the fold of barrettide C, a sample containing 2.2 mg/mL peptide was prepared for NMR spectroscopy and data were recorded at 600 MHz. Intriguingly, while the signal dispersion was excellent, the line width was broader than expected and far more signals than expected were present in the 2D TOCSY and NOESY data. A closer inspection and partial resonance assignments revealed that up to three similar, but subtly different spin systems were present for each amino acid. These spin systems represent conformational states that are in chemical exchange with each other, evident from intense cross-peaks between all involved resonances in both the NOESY and TOCSY data (Supporting Information Figure S2). While this resulted in a highly complex spectral appearance and prevented further in-depth analysis, we concluded the phenomena must be due to a dynamic multimerization process in which peptides are rapidly associating and disassociating. The NMR sample was consequently diluted 4-fold, and all data were rerecorded. Under these conditions, the lines were generally sharp, and all extra peaks had disappeared, allowing near complete resonance assignments using sequential assignment strategies.^48^ A full 3D structure was calculated based on interproton distance restraints, dihedral angles, and hydrogen bond restraints. The structure of barrettide C revealed a β-hairpin fold that is highly similar to the previously discovered and characterized barrettide A.^24^ Barrettide C contains two antiparallel β-strands, separated by a short loop, that are bridged by ladder-like disulfide bonds in a I–IV/II-III pattern (Cys5–Cys23 and Cys7–Cys18); in addition, a short α-helix is formed on the C-terminal side of the β-hairpin which is stapled to the N-terminal tail by the I–IV disulfide bond (Figure 3A). The structural statistics of barrettide C are presented in Table 2 and highlight the high quality of the structure and the agreement with the observed experimental data. The generated structures were of the 96th percentile of MolProbity scores, with 97% of residues falling within favored Ramachandran space and no outliers or poor rotamers present. Comparing the β-hairpin portion of barrettides A and C revealed a high structural similarity with a shared RMSD of 0.72 Å (Figure 3B). Despite barrettides A and C only differing by one residue within the ordered regions, barrettide C contains a short α-helix that was not observed in barrettide A. The data generated by barrettide C agree with the formation of this α-helical region with both temperature coefficients and preliminary calculations supporting the formation of the *i*–*i*_+4_ hydrogen bonds expected of an α-helix as well as TALOS-N predicting α-helical backbone angles. The structure has a significant hydrophobic patch, as shown in Figure 3C, and it is possible that it is responsible for the observed aggregation at higher concentration. Alternatively, two or more peptides may self-associate via their β-sheets to create intermolecular β-strand interactions and hydrogen bonds. The structure was deposited at PDB under 7SAG.

**Table 2:** NMR Distance and Dihedral Statistics for Barrettide C

Distance restraints	
sequential (|*i* – *j*| ≤ 1)	159
medium range (1 < |*i* – *j*| < 5)	26
long range (|*i* – *j*| ≥ 5)	56
hydrogen bonds	16 (8 H-bonds)
total	257
Dihedral angle restraints	
ϕ	23
ψ	25
total	48
Energies (kcal/mol, mean ± SD)	
overall	–992.94 ± 50.14
bonds	11.34 ± 0.80
angles	26.14 ± 2.44
improper	11.88 ± 1.48
dihedral	129.70 ± 1.37
van der Waals	–81.02 ± 5.90
electrostatic	–1091.30 ± 47.09
NOE	0.01 ± 0.01
constrained dihedrals	0.31 ± 0.20
Atomic RMSD (Å)	
mean global backbone (5–23)	0.76 ± 0.27
mean global heavy atoms (5–23)	1.35 ± 0.26
Molprobity	
clashes (>0.4 Å/1000 atoms)	4.60 ± 2.36
poor rotamers	0 ± 0
favored rotamers (%)	97.22 ± 3.15
Ramachandran outliers (%)	0 ± 0
Ramachandran favored (%)	97.41 ± 2.20
MolProbity score	1.36 ± 0.19
MolProbity score percentile	96.47 ± 3.10
Violations from experimental restraints	
NOE violations exceeding 0.2 Å	0
dihedral violations exceeding 2.0°	0

### Antifouling Assay and Biological Role

**Figure 4:**
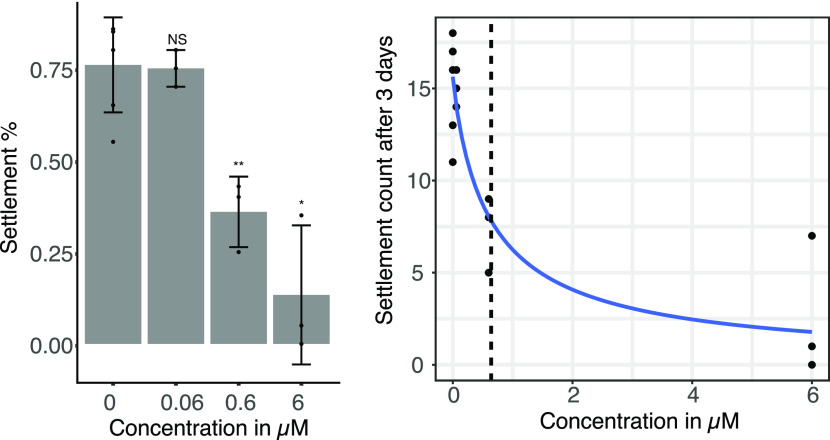
(Left) Larval settlement after 3 days of incubation with barrettide C. No mortality was observed. The experiments were performed in triplicate with *n* = 20 (±2) larvae per assay. Statistical evaluation was performed using a χ^2^ test comparing treatments to control. (Right) Dose–response curve of barnacle larval settlement after 3 days of incubation with barrettide C. The IC_50_ is indicated as a dashed line.

Prompted by the clean surface of the sponge, previous work on *G. barretti* compounds tested for biofouling inhibition.^19,49^ To follow up and further support the hypothesis that barrettide C belongs to a family of anti-biofouling peptides, we replicated the previous experimental setup.^19,49^ Settlement of bay barnacles was significantly inhibited at concentrations of 0.6 μM (χ^2^ test *p* = 6.255 × 10^–5^) and 6 μM (χ^2^ test, *p* = 7.206 × 10^–11^) barrettide C with an IC_50_ of 0.64 μM (Figure 4). These results were very similar to the activities of barrettides A and B^24^ and are in the range of commercially available but toxic antifouling compounds (EC_50_ TBTO (bis(tributyltin)oxide) 0.09 μg/mL or 0.15 μM and Sea-Nine 0.3 μg/mL or 1.23 μM).^50^ In contrast to previous work, we did not observe mortality during the experiment. At the highest treatment concentration, larval motor skills seemed impaired. While still occasionally moving their limbs, larvae did not move forward (or in any direction) in the Petri dishes.

Appropriate sequencing data allow one to investigate whether a certain compound is produced in response to an elicitor such as a competitor, ecological change, or another biological process. The number of transcriptomes is too small to draw any definite conclusions, but they allow for interesting speculation on the biology of the organisms and potential function of the peptides. For instance, the barrettide expression patterns for Gb01, Gb02, Gb03 and Gb04, Gb05, Gb06 are “mutually exclusive”. The first group of specimens were all collected in one locality, at one time, and they were not reproducing at the time of collection, whereas the latter group were all collected in different localities and season from the first group and two of them were reproducing (Gb04: female, Gb05: nonreproductive, Gb06: female).^51^ Thus, the barrettide pattern invites hypotheses about their expression as a function of biology (reproduction or general physiology, population differentiation) or the environment (nutrients, biotic/abiotic elicitors). From a phylogenetic perspective, the finding that *G. hentscheli*, the sister species of *G. barretti*,^52^ does not produce barrettides suggests these peptides may have appeared fairly recently in evolutionary terms. *G. barretti* and *G. hentscheli* diverged possibly during the glacial/interglacial cycles of the Quaternary period (approximately 2.6–0.0 Mya).^53^

In conclusion, we increase barrettide diversity by reporting five new barrettide sequences (C–G) discovered from high-throughput sequencing data of *G. barretti*. Four barrettides (C, D, F, G) are found in open reading frames. Barrettides C and D are further supported by mass spectrometric evidence from extracts of the sponge species. Regarding their distribution, the barrettides were found to be restricted to *G. barretti*, but there is evidence for similar hairpin peptides in other sponge species. We acknowledge that these questions are not exclusively addressed by sequencing data and can also be answered with equivalent MS-based experiments,^4^ but sequencing data are easily accessed and shared. The predicted barrettide C was synthesized and folded, and its native conformation was confirmed via coelution. Both NMR spectroscopy and antifouling assays yielded similar results to the previously described barrettides A and B. On the basis of the similarities in terms of sequence, structure, and activity among all predicted and investigated barrettides, we hypothesize that barrettides are a family of antifouling peptides. Evidence from sequencing data leads us to suggest the sponge as the producer of the barrettides. This work showcases how analytical chemistry and molecular biology go hand in hand, allowing for the expansion of peptide families. It also highlights the importance of including several samples and orthogonal methods for natural product discovery, as none of the data sets (genome, transcriptomes, metabolic profiles) on their own encompassed the barrettide’s diversity.

## Experimental Section

### NMR Spectroscopy

Lyophilized peptide (1 mg) was dissolved in 220 μL of H_2_O/D_2_O (9:1, v/v) at pH 5.15 (1.4 mmol/L). As this initial sample proved to be too concentrated, it was diluted 1:3 H_2_O/D_2_O (9:1, v/v) (0.35 mmol/L). This diluted sample was used to acquire one- and two-dimensional spectra (^1^H–^1^H TOCSY, ^1^H–^1^H NOESY, ^1^H–^15^N HSQC) at 298 K. An additional set of TOCSY spectra acquired at temperatures of 288, 293, 298, 303, and 308 K was used to obtain amide temperature coefficients. In addition, a series of ^1^H 1D/TOCSY spectra to monitor hydrogen–deuterium exchange as well as a ^1^H–^13^C HSQC spectrum were acquired at 298 K on a lyophilized peptide sample dissolved in 100% D_2_O. All data, including TOCSY (mixing time 80 ms), NOESY (mixing time 200 ms), and HSQC spectra, were recorded and processed using Topspin 4.1.1 (Bruker). Generally, 4096 data points were collected in the F2 dimension and 256 (128 complex) points in F1, with 512 increments of 8 scans over 11 194 Hz. NMR spectra were acquired on a Bruker Avance Neo 600 MHz (TCI (CRPHe TR-^1^H and ^19^F/^13^C/^15^N 5 mm-EZ)) spectrometer.

### Structure Calculation of Barrettide C

The structure of barrettide C was calculated using interproton distance restraints derived from the peak volumes of NOESY cross-peaks and dihedral ϕ (C_–1_–N–CA–C) and ψ (N–CA–C–N_+1_) backbone angles generated by torsion angle likelihood obtained from shift and sequence similarity (TALOS-N).^54^ Potential hydrogen bond donors were identified via the determination of backbone amide temperature coefficients. The chemical shift of the ^1^HN proton of each residue was plotted against temperature, with values > −4.6 ppb/K for the coefficient of the linear relationship taken as indicative of potential hydrogen bond donation.^55^ Hydrogen bond acceptors were determined during initial structure calculation. An initial set of structures (50) was generated through torsion angle dynamics and automated NOESY assignment using CYANA.^56^ The final 50 structures were annealed and refined in explicit water within CNS 1.21^57^ using protocols from the RECOORDscript database. All structures, containing no violations, were subjected to stereochemical analysis using MolProbity^58^ via comparison to structures published in the Protein Data Bank (PDB). From the final 50 structures, a set of 20 structures containing no violations, low energy, and best MolProbity scores was chosen. Final structures, restraints, and the chemical shift data for barrettide C have been submitted to the PDB (7SAG) and BRMB (30952).

### Previous Metabolic Profiles

Extracts from museum specimens identified by PC (deep sea NW Atlantic *G. atlantica* (UPSZMC 78293), *G. macandrewii* (UPSZMC 78255), *G. phlegraei* (UPSZMC 167249), *G. hentscheli* (UPSZMC 78266)) as well as samples Gb01, Gb02, and Gb03 were prepared following Cárdenas.^59^ In short, tissue subsamples were lyophilized and manually ground to a fine powder. A sample of 50 mg of powder was extracted using 4 mL of 60% MeCN and 0.1% formic acid (FA) in H_2_O on a shaking table for 2 h, the sample was precipitated at 3000 rpm for 10 min, the supernatant was collected, and the extraction was repeated with 30% MeCN and 0.1% FA in H_2_O and then with 0.1% FA in H_2_O. The combined extracts (12 mL) were diluted with 24 mL of 0.1% FA in H_2_O and desalted on a solid phase extraction (SPE) C_18_-EC column (Isolute, 10 mL, 500 mg). The columns had been solvated in methanol overnight, rinsed with 20 mL of 60% MeCN/0.1% FA in H_2_O, and equilibrated with 20 mL of 10% MeCN/0.1% FA in H_2_O prior to loading the diluted extracts. The columns were washed with 10 mL of 10% MeCN/0.1% FA in H_2_O, and less polar compounds were eluted with 5 mL of 60% MeCN/0.1% FA in H_2_O and lyophilized.

### UPLC-MS

For coelution experiments and acquisition of sponge metabolic profiles, lyophilized extract (and/or synthetic barrettide C) was dissolved in 10% MeCN/0.1% FA in H_2_O (4 μL/mg of sponge dry mass). Samples were analyzed with a Waters nanoAcquity ultra performance liquid chromatography (UPLC) system coupled to a Micromass Q-Tof micro mass spectrometer (MS), operated in positive mode and equipped with a nanolockspray interface. A BEH130 C18 nanoAcquity UPLC column (75 μm × 250 mm, 1.7 μm) was used for separation. A linear gradient elution from 1% to 90% B over 50 min followed by isocratic cleaning for 4 min and re-equilibration with 99% A was used, with mobile phase A (0.1% FA/0.05% MeCN in H_2_O) and B (0.1% FA in MeCN). The injection volume was 0.10 μL and the flow rate was 0.25 μL/min with a pressure between 7000 and 10 000 psi. Total analysis time was 75 min, and the *m*/*z* scan range was 50–1500. The spectra were analyzed with the program MassLynx (v. 4.1 Waters).

### Solid Phase Peptide Synthesis (SPPS)

Barrettide C was manually synthesized on 2-CTC resin (1.6 mmol/g) chlorinated overnight with 120 μL of thionyl chloride, 200 μL of pyridine, and approximately 4 mL of CH_2_Cl_2_. The first amino acid (0.632 mmol) dissolved in CH_2_Cl_2_ was coupled with 7 equiv of *N*,*N*-diisopropylethylamine (DIPEA) overnight. The resin was washed and dried, and the new loading was estimated by weight increase (0.85 mmol/g resin). The synthesis procedure included deprotection with 20% piperidine twice, for 1 and 20 min, respectively, and amino acid coupling using 5 equiv of AA, 10 equiv of 1 M DIC, and 10 equiv of 2 M OxymaPure (30 min × 2). Ser and Gly in position 18 and 19, respectively, were replaced by coupling the Fmoc-Ser(tBu)-(Dmb)Gly-OH dipeptide to improve synthesis efficiency. The deprotected peptide was cleaved off the resin by incubating in a cleavage mixture with trifluoroacetic acid (TFA), triisopropylsilane (TIPS), and H_2_O (9.5:0.25:0.25, v/v) for 2 h. The crude peptide was dissolved in 20% MeCN in MQ-H_2_O and purified on an RP-HPLC system (Shimadzu LC-20) with a preparative column (Jupiter 15 μm, C18, 300 Å, 250 × 21.2 mm, Phenomenex) on a linear gradient of 5–90% solvent B (MeCN with 0.05% TFA). The presence of the peptide in the fractions was assessed by MS (ThermoQuest Finnegal LCDeca). Fractions containing the peptide were lyophilized then oxidatively folded with a buffer of 1 M NH_4_HCO_3_, 4 mmol of glutathione disulfide (GSSG), 20 mmol of glutathione (GSH), and peptide dissolved in 5 mL of MQ-H_2_O with stirring overnight. The peptide was fractionated on a semipreparative column (Jupiter 5 μm, C18, 300 Å, 250 × 10 mm Phenomenex). Fractions with folded peptide were detected with MS as before, and their purity was assessed on an RP-HPLC (Shimadzu LC-10) with an analytical column (XSELECT CSH 130 C18 2.5 μm, 4.6 × 150 mm column XP, Waters). Peptide purity was assessed to be >95% by UV (215 nm). Fractions containing the folded peptide were lyophilized.

### Antifouling Assay

Biofouling inhibition was tested with a similar experimental setup to that for barrettides A and B.^19,24,49^ In short, 20 (±2) cyprid larvae of the bay barnacle *Amphibalanus improvisus* were incubated in 9.9 mL of filtered seawater (FSW) + 100 μL of additive, the additive being either 100 μL of FSW, 100 μL of sterile filtered H_2_O (controls), or 100 μL of peptide dissolved in MQ-H_2_O (treatment). Peptide concentrations tested were 0.06, 0.6, and 6 μM, all experiments were performed in triplicates, and the settling was assessed after 3 days of incubation. Statistical analyses were produced in R^60^ and included χ^2^ test, ggplot2 for visualizations,^61^ and the package drc^62^ for dose–response curve and IC_50_ calculations.

### Sample Description

Three *G. barretti* specimens (Gb01, Gb02, Gb03) were collected West of Yttre Vattenholmen (58.876233, 11.101483), Kosterfjord, Sweden, on May 4, 2016, at 84–96 m depth, using a remote operated vehicle on board the R/V *Nereus*. Samples were identified on board by PC, and subsamples for transcriptomics were flash-frozen on board in liquid N_2_ then kept at −80 °C until RNA extraction; subsamples were also frozen for chemistry. A subsample of Gb01 was frozen and used to extract high molecular weight DNA for whole genome sequencing. Vouchers of Gb01, Gb02, and Gb03, stored in 96% EtOH, have been deposited at the Zoological Museum of Uppsala (UPSZMC), Sweden, under museum numbers UPSZMC 184975, 184976, and 184977, respectively. Another four *G. barretti* transcriptomes were derived from specimens collected during the deep-sea expedition GS2017110 on the R/V *G.O. Sars*. Of those, one specimen came from Sula Reef, Norwegian Sea (64.0749, 8.0270, July 23, 2017, ROV dive 6, 267 m) and three specimens came from Tromsøflaket, Barents Sea (71.5870, 21.3750, August 3, 2017, trawl 1, 333 m); these four specimens were identified by Hans Tore Rapp, and vouchers are stored in the Riesgo lab at −80 °C under ROV6#3Gb (Gb04), trawl1#5Gb (Gb05), trawl1#6Gb (Gb06), and trawl1#8Gb (Gb07) (Natural History Museum, London, UK). Raw RNA sequencing data were obtained for these seven individuals of *G. barretti* (Gb01, Gb02, Gb03, Gb04, Gb05, Gb06, Gb07); for a complete description of the methods for the transcriptome sequencing, see Koutsouveli et al.^51^

### Data Preparation

For *G. barretti* transcriptomes, raw data were quality checked with fastQC v.0.11.9.^63^ Transcriptomes were assembled with Trinity v.2.9.1/v.2.11. In parallel, the raw reads were also processed with trimmomatic v.0.36^64^ and downsampled with khmer v.2.1.1^65^ normalizing by median. The assemblies were cross-checked by Transrate v.1.0.1^66^ with the trimmed and downsampled reads, and only the good transcripts were used for BLAST searches. The number of genes was approximated by counting the unique Trinity sequence cluster IDs. The metagenome^29^ is Roche 454 data and was also assembled with Trinity^67^ but without any further processing. Readily assembled sponge transcriptomes (*n* = 63) were provided by collaborators or downloaded from open data repositories (Supporting Information Table S1).

### BLAST Searches of Transcriptomes for Similar Sequences

For all sequencing data resources, local BLAST databases were built and searched (tblastn) with the amino acid sequences of barrettide A and B as queries. Tabular results were concatenated separately for *G. barretti* and all other sponges, asterisks and dashes were removed from the amino acid sequence of hits, and only those fitting the cysteine framework of barrettides were retained (Supporting Information Tables S2, S4).

### Transcriptome Annotation

Transcriptome annotation was performed for one transcriptome (Gb01) following the Trinotate workflow.^68^ Open reading frames were extracted from the transcriptome with TransDecoder v. 5.5.0 (https://github.com/TransDecoder/TransDecoder). The output was submitted to the online portal GhostKOALA v. 2.2 (https://www.kegg.jp/ghostkoala/), and open reading frames were annotated against KEGG database ‘c_family_euk+genus_prok’.^69^

### Manual Sequence Analysis

Barrettide hit sequences were manually extracted from the assemblies and barrettides present in ORFs were identified with https://web.expasy.org/translate/. The translated ORFs were submitted to SignalP-5.0 server^31^ (http://www.cbs.dtu.dk/services/SignalP/) for prediction of the signal peptide sequence. Translated barrettide ORFs were also submitted to a webtool (https://web.expasy.org/peptide_cutter/) for prediction of cleaving enzymes.

## Supporting Information Available

File S1: Information on Tables S1–S6 and Figures S1, S2 (PDF)
File S2: Tables S1–S6 (XLSX)
