# Supplementary material for: Barrettides: A Peptide Family Specifically Produced by the Deep-Sea Sponge Geodia barretti
Source: J Nat Prod. 2021 Dec 7;84(12):3138–46. doi: 10.1021/acs.jnatprod.1c00938 (PMC8713285; doi:10.1021/acs.jnatprod.1c00938)
Supplement: Supplementary file 1 — np1c00938_si_001.pdf [file np1c00938_si_001.pdf]

Supporting Information

Barrettides: A Peptide Family Specifically Produced by the Deep-Sea Sponge *Geodia barretti*

Karin Steffen†, Quentin Laborde†, Sunithi Gunasekera†, Colton D. Payne‡, K. Johan Rosengren‡, Ana Riesgo¶§, Ulf Göransson†, and Paco Cárdenas†.

† Pharmacognosy, Department of Pharmaceutical biosciences, Biomedical Centre, Uppsala University, Husargatan 3, 751 23 Uppsala, Sweden.

‡ School of Biomedical Sciences, The University of Queensland, Brisbane, QLD 4072, Australia.

¶ Department of Life Sciences, The Natural History Museum, Cromwell Road, London SW7 5BD, United Kingdom.

§ Department of Biodiversity and Evolutionary Biology, Museo Nacional de Ciencias Naturales – CSIC, Calle José Gutiérrez Abascal 2, 28006, Madrid, Spain.

Supplementary Tables

All six supplementary tables are combined into one Excel file and provided separately.

- Table S1: List of all sequencing ressources (transcriptomes, metatranscriptome and genome) and accession numbers, if available.
- Table S2: List of BLAST hits in transcriptomes, metatranscriptomes and the draft genome assembly of *G. barretti*.
- Table S3: Nucleotide sequences of barrettides.
- Table S4: List of BLAST hits in transcriptomes other than *G. barretti*.

- Table S5: Amino acid analysis and peptide sequencing information for barrettide A from Carstens et al., 2015.
- Table S6: Amino acid analysis and peptide sequencing information for barrettide B from Carstens et al., 2015.

Supplementary Figures

- Figure S1: Steffen et al. (*unpublished*) sampled *Geodia barretti* along a depth range from 407 to 1462 m in the Davis Strait between Canada and Greenland. The signal of barrettide C recorded by UPLC-HRMS was not affected by sample depth.
- Figure S2: Spectral complexity of Barrettide C when studied at high concentration versus low concentration.

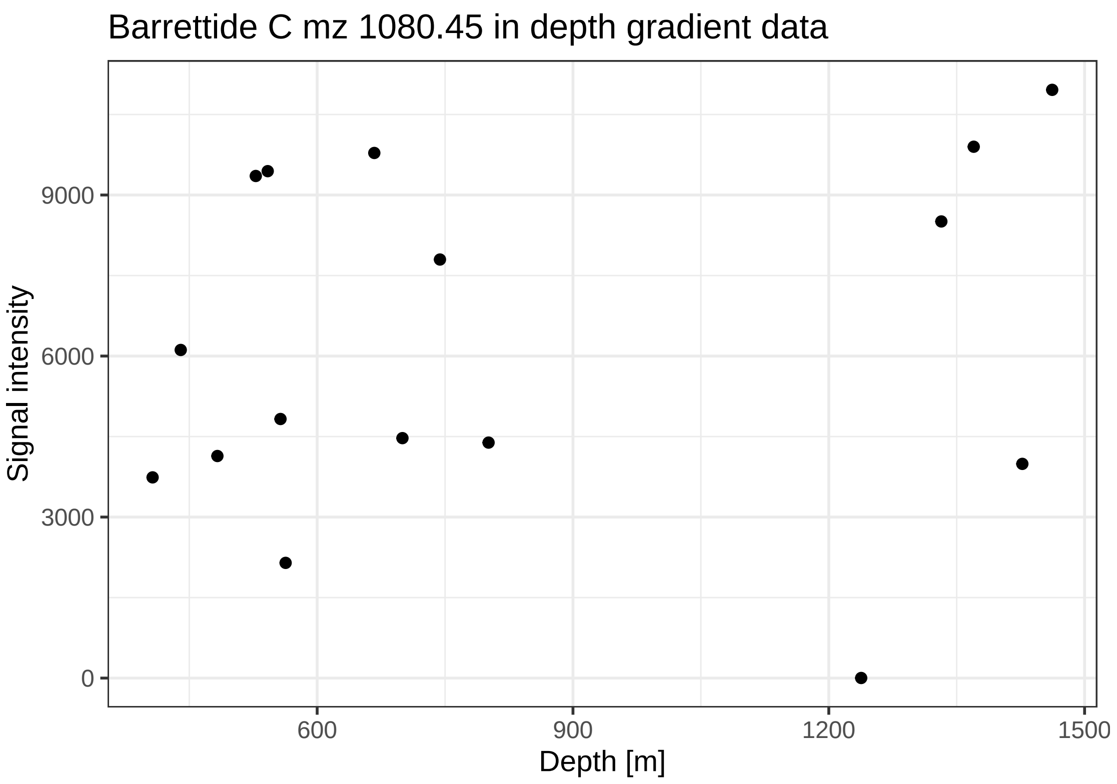

Figure S1: Barrettide C ($[M+3]^{3+}$) in *G. barretti* sampled along a depth gradient in the Labrador Sea.

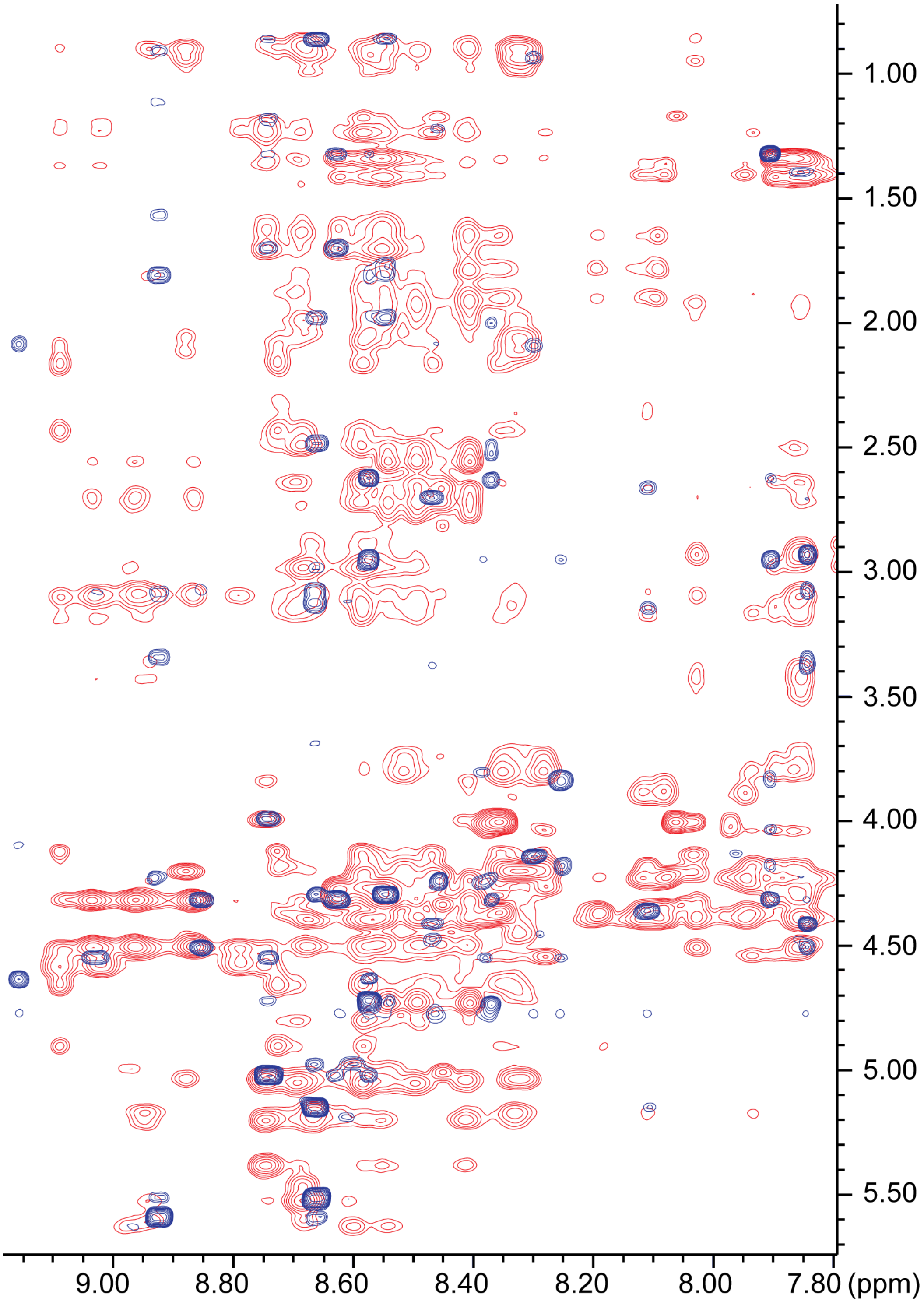

Figure S2: Overlay of the fingerprint region of Barrettide C. The NOESY spectrum of Barrettide C at a high concentration is shown in red and is overlaid with the low concentration spectrum in blue. Recorded at 600 MHz and 298 K.
